# Acetaminophen-induced apoptosis: Facts versus fiction

**Published:** 2020-08-01

**Authors:** Hartmut Jaeschke, Anup Ramachandran

**Affiliations:** Department of Pharmacology, Toxicology and Therapeutics, University of Kansas Medical Center, Kansas City, KS, USA

**Keywords:** acetaminophen, apoptosis, caspases, DNA fragmentation, drug hepatotoxicity, necrosis

## Abstract

An overdose of the widely used analgesic acetaminophen (APAP) is the most common cause of acute liver failure in the western world and hence is a clinically significant problem. Thus, mechanisms of APAP-induced hepatotoxicity have been the focus of extensive investigation for decades and it was established that APAP induces hepatocyte cell death by necrosis. Although APAP-induced necrosis shares some features of apoptosis induced by the intrinsic pathway, apoptotic cell death in this context was ruled out due to the absence of caspase activation and lack of protection by caspase inhibitors and missing morphological characteristics of apoptotic cells. Deeper mechanistic understanding of the cell death process after APAP in recent years has now revealed that cells die by programmed necrosis and apoptosis is not a relevant mode of cell death in this context. Hence, it is alarming to note that an increasing number of studies are being published purporting to indicate that APAP induces apoptotic cell death. These papers broadly measure “apoptotic markers” with questionable specificity such as Bax, Bcl-2 and caspase-3 protein expression, or use the terminal deoxynucleotidyl transferase dUTP nick end labeling assay as basis for the conclusion that there is apoptosis after APAP overdose. The misguided use of these apoptosis parameters in correlative studies without context or scientific rationale confuses the field and threatens to undo decades of careful mechanistic investigation into this topic. This review examines this emerging problem in detail and recommends approaches to correct it.

**Relevance for Patients:**

Hepatotoxicity and acute liver failure caused by an acetaminophen overdose is a serious clinical problem in western countries. Understanding the mode of cell death and the signaling pathways involved is critical for developing new therapeutic approaches. Recent trends to claim that apoptosis is a relevant mode of cell death in acetaminophen hepatotoxicity are not justified by sound scientific data and will not lead to effective new drug development.

## 1. Introduction

Cell death is a key event of almost all disease processes. Until the 1990s, necrosis was considered the dominant mode of cell death involving a catastrophic stress event that causes the cell to die. Only certain aspects such as reactive metabolite formation and protein binding, excessive cellular Ca^2+^ accumulation, oxidant stress and lipid peroxidation, or adenosine triphosphate (ATP) depletion were investigated as mechanisms of cell death [1]. However, no necrotic signaling pathways were considered at that time. In the early 1990s, apoptotic cell death became popular not only because it was a new and exciting topic but also because for the first time there were intracellular signaling pathways of cell death to be explored. Therefore, apoptotic cell death mechanisms were intensely investigated and by the year 2000, apoptosis was identified as a dominant mode of cell death in virtually all liver diseases [2]. However, we have previously argued that cell death in a lot of these processes, including hepatic ischemia-reperfusion injury [3,4] and obstructive cholestasis [5,6], was incorrectly labeled as apoptosis but was in fact necrotic cell death.

Despite the popularity of apoptosis in general, in acetaminophen (APAP) hepatotoxicity most leading groups in the field at that time including Steven Cohen [7], Jack Hinson [8], George Corcoran [9], Sid Nelson [10], Debra Laskin [12], and others stayed with necrosis as the mode of cell death. Only a few isolated papers claimed a role for apoptosis in APAP hepatotoxicity between 1995 and 2003 [12,13]. During that time, we carefully evaluated the morphological features of APAP-induced cell death, caspase activation and the effect of caspase inhibitors in this model [14,15] and demonstrated unequivocally that the mode of cell death is not apoptosis but oncotic necrosis. In addition, we could show that some of the claims for caspase inhibitors being protective [13,16] were based on solvents such as dimethyl sulfoxide (DMSO), which inhibits cytochrome P450, rather than caspase inhibition [17,18]. Thus, the issue seemed to be resolved, i.e., there was no credible evidence that apoptosis is a relevant mode of cell death during APAP-induced liver injury *in vitro* or *in vivo*.

However, during the last decade there has been an increasing number of papers published that claim apoptotic cell death being important in the pathophysiology. Based on the Web-of-Science citation index, a search of “acetaminophen” and “apoptosis” showed two papers published in 1995 but 115 papers for 2019, i.e., a >50-fold increase. In contrast, “acetaminophen” and “necrosis” indicated 84 papers in 1995 and 157 in 2019, i.e., a less than 2-fold increase. In fact, 70% of all APAP papers that considered apoptosis were published in the past 10 years, i.e., well after the peak of apoptosis research occurred for virtually every other disease. The questions arise: Was anything missed in these earlier studies? Were new aspects of apoptosis discovered that fundamentally changed the mechanisms of APAP-induced cell death? This review explores the reasons for the current development in this field and provides some suggestions to resolve the current dilemma.

## 2. Acetaminophen Hepatotoxicity – The Clinical Problem

APAP is a widely used analgesic and antipyretic drug, which is safe at therapeutic doses but can cause dose-dependent liver injury and even liver failure after an overdose [19]. Clinical studies have shown that more than 30,000 people are hospitalized due to overdose on APAP-containing drugs per year in the US and 300-500 people die from APAP-induced acute liver failure [20,21]. These deaths represent almost 50% of all acute liver failure patients in the US; many western countries deal with similar numbers indicating that APAP overdose is a significant clinical problem [22]. N-acetylcysteine (NAC) was introduced as an effective clinical antidote [23,24] based on early mechanistic insight through animal studies [25,26]. NAC is highly effective in preventing liver injury and liver failure in early presenting patients, i.e., when NAC is administered within 8 h after the overdose, but loses efficacy at later times [27,28]. Since a significant number of patients take a very large overdose, and/or present late for treatment, there is still the need to develop additional therapeutic options through better understanding the signaling mechanisms of cell death [29].

## 3. Signaling Mechanisms of Apoptotic Cell Death

**Figure 1:**
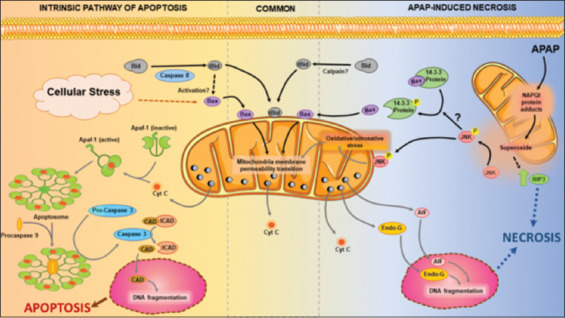
Common pathways shared between intrinsic induction of apoptosis and analgesic acetaminophen (APAP)-induced programmed necrosis and mechanistic distinctions: Although APAP-induced programmed necrosis has some common features with intrinsic apoptosis; they are mechanistically distinct and ultimately produce different modes of cell death. The intrinsic pathway of apoptosis is activated by cellular stress which activates Bax, which may also be induced by caspase 8 mediated tBid formation from Bid. Bax and tBid translocation to the mitochondria induces the mitochondrial membrane permeability transition (MPT) leading to release of inter-mitochondrial membrane proteins such as cytochrome c. Cytochrome c interaction with APAF1 results in its activation and formation of the apoptosome platform which facilitates caspase 9 mediated conversions of pro-caspase 3 to its active form. Active caspase-3 cleaves inhibitor of caspase-activated DNase to release caspase-activated DNase, which translocates to the nucleus and initiates nuclear DNA fragmentation, resulting in apoptosis. While APAP-induced hepatocyte death also induces Bax and tBid mitochondrial translocation as well as the MPT and cytochrome c release, significant differences in upstream mechanisms exist. APAP overdose results in generation of the reactive metabolite NAPQI which forms mitochondrial protein adducts which inhibit the electron transport chain and result in excessive release of superoxide radicals. This then activates JNK in the cytosol, which translocates to the mitochondria and may also facilitate Bax translocation by phosphorylation of its binding partner 14-3-3 in the cytosol. Mitochondrial JNK translocation amplifies the oxidative and nitrosative stress, which along with Bax translocation results in induction of the mitochondrial permeability transition. This releases proteins such as cytochrome c, apoptosis inducing factor (AIF), and endonuclease G into the cytosol. However, the probable lack of adenosine triphosphate prevents apoptosome formation and translocation of AIF and endonuclease G to the nucleus then results in DNA fragmentation. This, along with upregulation of the receptor interacting kinase 3, a molecular switch for programmed necrosis, then leads to necrotic cell death. This figure includes a modified template from Servier Medical Art, which is licensed under a Creative Commons Attribution 3.0 generic license; https://smart.servier.com.

**Figure 2:**
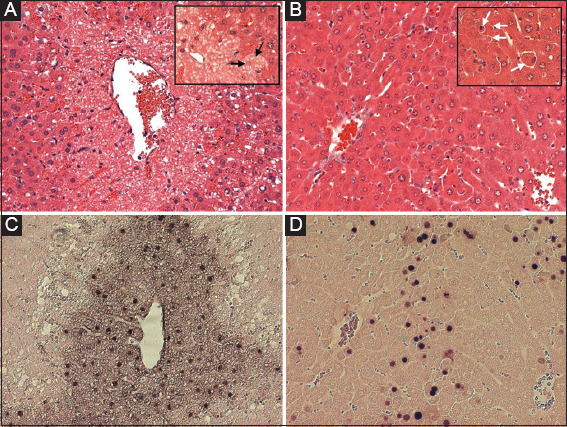
Fasted C57BL/6J mice were treated with analgesic acetaminophen (APAP) (300 mg/kg; 6h) (A and C) or galactosamine/endotoxin (700 mg/kg/100 mg/kg; 5 h) (B and D). Hematoxylin and eosin-stained tissue sections show necrotic cells with vacuolization, cell swelling, and nuclear disintegration after APAP overdose (A; black arrows). Sections of Gal/ET-treated mice show nuclear condensation, cell shrinkage, and formation of apoptotic bodies (B; white arrows). Sections stained with the TUNEL assay show nuclear and cytosolic staining in necrotic cells (C) and distinct nuclear staining in apoptotic cells (D). × 200; × 400 (inserts). Reproduced with permission from [18].

To be able to determine whether cell death is mediated through apoptosis, a brief review of the relevant intracellular signaling pathways is needed (Figure 1) [30,31]. Apoptosis can be induced in hepatocytes by ligands such as tumor necrosis factor (TNF)-a or Fas-Ligand, which activate the TNF- or Fas receptor, respectively (extrinsic pathway). Ligand binding results in trimerization of the receptor with assembly of the death-inducing signaling complex, which activates initiator caspases, for example, pro-caspase-8. Although the active caspase-8 can directly cleave pro-caspase-3 and trigger downstream events of apoptosis signaling, in hepatocytes, this requires amplification of the signal through the mitochondria (type II cell). Caspase-8 cleaves Bid and the truncated form of Bid translocates to the mitochondria and inserts into the outer mitochondrial membrane and forms pores with other pro-apoptotic Bcl-2 family members. Besides the initiation of apoptosis by this extrinsic pathway, cellular stress can lead to the activation of apoptosis through the intrinsic pathway. This involves mainly translocation of pro-apoptotic Bcl-2 family members such as Bax to the mitochondria and the triggering of mitochondrial outer membrane permeabilization. Independent of the initiation signal, the increased permeability of the outer mitochondrial membrane causes the release of intermembrane proteins such as cytochrome c and Smac/Diablo. Cytochrome c forms the apoptosome, together with apoptotic protease activating factor 1 (APAF1), pro-caspase-9, and ATP, which results in caspase-9 activation. The active caspase-9 cleaves pro-caspase-3 resulting in the activation of the downstream apoptotic signaling pathways. Smac/Diablo release into the cytosol causes the inactivation of the inhibitor of apoptosis proteins, whose function is to prevent the accidental activation of caspases. Once effector caspases such as caspase-3 are activated, they cleave the inhibitor of caspase-activated DNase (ICAD) and liberate the actual enzyme caspase-activated DNase (CAD), which then degrades DNA in the nucleus into internucleosomal fragments of roughly 180 base pairs and multiples thereof, resulting in a characteristic DNA ladder. The morphology of a cell undergoing apoptosis is characterized by cell shrinkage, chromatin condensation, and apoptotic body formation (Figure 2B). Biochemically, the main parameter specific for apoptosis is the extensive activation of caspases identified by increase in enzyme activity or by western blotting showing the cleavage of the pro-caspase into the active fragments. In addition, apoptotic cell death is effectively inhibited by caspase inhibitors, which are generally suicide substrates. Unfortunately, virtually all other parameters described above are not specific for apoptotic cell death and cannot be used to reliably identify apoptosis as the exclusive mode of cell death.

## 4. Signaling Mechanisms of APAP-induced Cell Death: Programmed Necrosis

The intracellular signaling mechanisms of acetaminophen-induced liver injury have been reviewed in detail (Figure 1) [32-35]. Although the majority of any APAP dose is metabolized by phase II conjugation reactions, a portion is metabolized by cytochrome P450 enzymes, especially Cyp2E1, to form a reactive metabolite, N-acetyl-p-benzoquinone imine (NAPQI), which is conjugated by hepatic glutathione (GSH) [33]. After an overdose, hepatic GSH levels are rapidly depleted and NAPQI binds to sulfhydryl groups of proteins. Mechanistically the most important protein binding occurs to mitochondrial protein resulting in a moderate oxidant stress that triggers activation of redox-sensitive mitogen-activated protein kinases, which ultimately causes the phosphorylation of c-jun-N-terminal kinase (JNK) [34,35]. P-JNK translocates to mitochondria and aggravates the mitochondrial oxidant stress and peroxynitrite formation leading to the opening of the mitochondrial membrane permeability transition pore (MPTP) [36,37]. In addition to leading to the breakdown of mitochondrial membrane potential and cessation of ATP synthesis, MPTP opening also causes matrix swelling and rupture of the mitochondrial outer membrane. Both an earlier translocation of Bax to the outer membrane and the later MPTP and rupture of the outer membrane cause the release of intermembrane proteins such as cytochrome c, Smac/Diablo, endonuclease G, and apoptosis-inducing factor (AIF) [38]. Interestingly, despite the mitochondrial release of cytochrome c and Smac/Diablo, no activation of the apoptosome and caspases can be detected. This is not caused by a direct binding of NAPQI to sulfhydryl groups of pro-caspases [14] but more likely due to significant ATP depletion [39]. In contrast, endonuclease G and AIF released from mitochondria translocate to the nucleus and cause nuclear DNA fragmentation [40]. Ultimately, the severe mitochondrial dysfunction and the extensive nuclear DNA fragmentation are responsible for cell necrosis characterized by extensive cell and organelle swelling, karyorrhexis, and release of cell contents (Figure 2A) [15]. Although additional proteins beyond what has been so far described may be involved in cell death, the general conclusion remains that this is the fundamental framework of APAP-induced cell death representing oncotic necrosis [41]. Based on the critical importance of drug metabolism and JNK-mediated mitochondrial oxidant stress, novel therapeutics are being clinically tested [29].

## 5. Misinterpretation of “Apoptosis Parameters” in APAP Hepatotoxicity

The conclusions by many recent studies that there is substantial apoptotic cell death in the murine model of APAP hepatotoxicity, assumes that certain parameters are indicative of apoptosis. The most prevalent misconceptions are discussed.

### 5.1. Bax and Bcl-2 mRNA and protein expression

The most used parameters are the mRNA or protein expression of pro-apoptotic Bax and anti-apoptotic Bcl-2 measured with reverse transcription-polymerase chain reaction or western blotting, respectively, in the whole liver homogenate. According to these studies, there is always a low baseline expression of Bax mRNA and protein in controls, a substantial increase after APAP, and a significant decrease with whatever intervention is being tested [42-50]. In contrast, Bcl-2 seems to be always high in controls, lower with APAP alone, and again higher but not totally back to baseline levels with the intervention [42-50]. In some cases, Bax and Bcl-2 protein changes are also shown selectively around the central vein area by immunohistochemistry [42,49,51]. In addition, the Bax-to-Bcl-2 ratio is gaining popularity as another parameter to be displayed [42,47,48]. Together, these data are considered a strong argument for the conclusion of apoptotic cell death during APAP toxicity. However, there are many arguments why this is not justified by the data and is actually incorrect. Bax protein is expressed at baseline in every cell including all hepatocytes. To promote cell death, Bax protein does not have to be newly synthesized but needs to translocate from the cytosol to the mitochondria to form pores and increase the mitochondrial outer membrane permeability. This has been shown for APAP-induced necrosis where the Bax pore facilitates the early release of AIF and endonuclease G for DNA fragmentation [38]. Although there is also some release of cytochrome c and Smac/Diablo, there is never any caspase activation [38]. In addition, the effect of Bax on DNA fragmentation and cell death is only transiently relevant during the very early phase of the injury being replaced by release of these intermembrane proteins due to mitochondrial matrix swelling and rupture of the outer membrane during the latter phase [38]. In contrast, Bcl-2 is generally not expressed in hepatocytes at baseline [10,52-54] but can be induced during APAP toxicity [54]. In addition, if Bcl-2 expression is protective as assumed by all these studies, the question is why the alleged initial high baseline expression in controls does not prevent apoptosis when treated with APAP, but a return of Bcl-2 levels to 50% of control values by a therapeutic intervention is surprisingly effective in protection. Of course, this makes no sense and it remains a mystery as to what is actually measured as Bcl-2 protein by these studies. Interestingly, Bcl-2 overexpressing mice do not seem to be protected at all but suffer increased APAP-induced liver injury [10]. Together, the assessment of Bax and Bcl-2 protein expression as performed in most of these intervention studies and their interpretation and conclusions are highly questionable as there are serious design flaws in these correlation studies, which do not test causality.

### 5.2. Terminal Deoxynucleotidyl Transferase dUTP Nick End Labeling (TUNEL) assay

The TUNEL assay is a widely used method to detect DNA fragmentation, i.e., DNA double strand breaks. Although originally assumed to be specific for apoptosis, it was already shown 25 years ago that this is not the case; this assay detects DNA fragmentation during apoptosis, necrosis, and autolytic cell death [55]. We have shown that most dying cells during APAP hepatotoxicity are TUNEL-positive (Figure 2C) [14,15]; the DNA fragmentation is caused by mitochondrial endonucleases which are released and translocate to the nucleus [40]. Importantly, preventing mitochondrial dysfunction by scavenging peroxynitrite by newly synthesized GSH [56,57], preventing peroxynitrite formation with a mitochondria-targeted SOD mimetic [58] or suppressing superoxide formation with a JNK inhibitor [59] all eliminate nuclear DNA fragmentation during APAP overdose. In striking contrast, a pan-caspase inhibitor, which effectively prevents activation of CAD and eliminates DNA fragmentation measured by the TUNEL assay during TNF-induced apoptosis [60], has no effect on APAP-induced DNA damage [57]. Because of these fundamentally different mechanisms between CAD-induced and AIF/endonuclease-induced DNA fragmentation, the DNA fragments generated are different in size between TNF-induced apoptosis and APAP-induced necrosis [61]. This results in a fundamentally different staining pattern with the assay [15,18]. Whereas, apoptotic cells have a very distinct nuclear staining, necrotic cells show nuclear staining and staining of the cytosol (Figure 2C and D) [15,18]. Thus, use of the TUNEL assay with proper positive controls for apoptosis clearly demonstrates that APAP-mediated DNA fragmentation is different from that seen during apoptosis.

### 5.3. Caspase activation and effect of caspase inhibitors

Caspases are initiators and executioners of apoptosis signaling and there is consensus that activation of caspases such as caspase-3 and others are specific indicators of apoptotic cell death [62,63]. Therefore, when we and others started to assess if there was apoptosis in APAP toxicity, caspase activities were measured. Consistently, no elevated caspase enzyme activities were found [10,13-15]. In contrast, during TNF- or Fas-induced apoptosis affecting overall less hepatocytes than APAP toxicity, there was a 50-100-fold increase of caspase-3 activity [60,64]. During APAP hepatotoxicity, no caspase-3 processing was detected by western blotting [14,15]. It has to be emphasized that the western blots were run to detect the uncleaved pro-caspase-3 and the fragments on the same gel and that in addition to APAP samples also negative (untreated) and positive (TNF-apoptosis) controls were included [14,15]. This clearly indicated that consistent with the enzyme activity measurements, there is no evidence for any relevant activation of caspases during APAP toxicity. This was further supported by experiments where highly effective pan-caspase inhibitors were used. These inhibitors eliminated caspase activity after Fas- and TNF-induced apoptosis [60,64,65] but had no effect on APAP-induced toxicity [14,15,17,57,66]. These biochemical data together with the absence of morphological evidence of apoptosis (Figure 2) [15] led to the very strongly supported conclusion that apoptotic cell death is not a relevant contributor to APAP-induced liver injury.

Initially, only two studies disagreed. The first one did also not find any increase in caspase activity but reported that a pan-caspase inhibitor eliminated the injury [13]. Although the lack of caspase activation was consistent with our findings, it made no sense that a caspase inhibitor can protect because these inhibitors are suicide substrate, which require an active enzyme. As the authors of this paper used the same APAP dose and the same caspase inhibitor [13] as we did previously [14,15] except as a pretreatment, we repeated their experiments and could unequivocally demonstrate that the protective effect of the pan-caspase inhibitor pretreatment was due to the solvent DMSO [17], which is a potent P450 inhibitor [67]. A second study claimed to use a novel pancaspase inhibitor dissolved in an unspecified “solvent” protected against APAP toxicity [16]. However, the vendor website clearly indicated that this inhibitor was only soluble in DMSO and given the pre-treatment schedule and dose, the amount of DMSO used in this experiment and the absence of a proper solvent control (APAP+DMSO) led us again to the conclusion that the solvent and not the inhibitor were responsible for the protection [18]. Thus, there are no credible reports in the literature that showed evidence for caspase-mediated apoptosis in APAP toxicity.

More recently, some studies using western blots showed an induction of caspase-3 protein expression during APAP overdose, which can be prevented with some herbal or other interventions [46,48,50]. However, assessing caspase protein induction is meaningless as every cell has enough pro-caspases to execute apoptosis when properly stimulated. Furthermore, it would be inefficient for a cell to have to transcriptionally activate caspase gene expression and protein synthesis before it can undergo apoptosis.

Some investigators have also shown an increase in cleaved caspase-3 and other caspase fragments [49,50]. However, the absence of the pro-enzyme and of any positive control makes these types of blots difficult to interpret. The fact that control samples show extensive cleaved fragments indicates that these blots are severely overexposed [49,50]. Densitometric analysis indicated an 80-150% increase of caspase cleavage [49,50], which would be quantitatively insufficient to explain cell death in this model. As mentioned, a 3000-5000% increase in caspase activities would be expected [60,64]. Furthermore, when the changes in TUNEL-positive cells in these studies show that 80% of hepatocytes are allegedly undergoing apoptosis, and this correlates with less than a doubling of the caspase-3 cleavage [50], there is a gross quantitative mismatch between these apoptosis parameters which make no sense if actual induction of apoptosis was taking place. Similarly, there are antibodies that may stain for cells that are positive for cleaved caspase-3 [68]. However, the quality of staining is generally very poor and the number of cells stained is very limited. In addition, the absence of a positive control for apoptosis and other control experiments do not instill a lot of confidence in the validity of these data. Importantly, none of these studies included a specific intervention against caspases, which makes these studies only correlative without evidence that any of these changes in caspases can be responsible for the injury.

Given the discussed caveats with many of these parameters and the concerns with their conclusions in these correlative studies, on the one hand, and the extensive studies directly testing the hypothesis of apoptotic cell death, on the other hand, it seems very obvious that apoptosis is not a relevant component of the mechanism of APAP hepatotoxicity.

## 6. Apoptosis and Secondary Necrosis

The original concept of apoptotic cell death as a mechanism to remove cells during development or aging generally involves individual cells where the process of apoptosis can be completed, i.e., the cell disintegrates into apoptotic bodies, which are removed by phagocytes or neighboring cells. However, under pathophysiological conditions many cells may be affected, and the process is accelerated, which means that the cell may not be able to maintain their ATP levels and the apoptotic process deteriorates into secondary necrosis. Under these conditions, the cell membrane integrity is lost and the cell behaves like a cell undergoing necrosis with one exception: There are still very high caspase activities in the cell and a pan-caspase inhibitor will eliminate the initial apoptosis and prevent the secondary necrosis [6,64]. Given these characterizations, it is highly unlikely that APAP-induced cell death is apoptosis deteriorating to secondary necrosis.

A case where apoptosis can become detectable after exposure to APAP is when the necrotic process is inhibited. This was shown in primary mouse hepatocytes when the MPTP opening was prevented by glycine and fructose treatment and 10 h later apoptosis developed [37]. This was confirmed *in vivo* in mice when long after the effective protection against APAP-induced mitochondrial oxidant stress by Mito-TEMPO a limited number of cells were undergoing apoptosis [69]. In both cases, the programmed necrotic pathway triggered by APAP overdose was inhibited by blocking downstream events. However, this means that the upstream stress of reactive metabolite formation, GSH depletion, and protein adducts formation is still present and some cells do not tolerate this stress and eventually undergo apoptosis. It is critical to distinguish these secondary effects from the primary necrotic process induced by APAP.

However, why does APAP overdose induce early signals of mitochondrial apoptosis such as Bax translocation and cytochrome c release without activation of caspases if cells were programmed to die by necrosis? The evidence of these early apoptotic features most likely merely illustrates the redundancy of cell death mechanisms within hepatocytes. This concept of an ancestral cell death mechanism such as necrosis which subsequently incorporates features of apoptosis to probably gain selective evolutionary advantage under certain conditions has been recognized for a number of years [70]. Here, the process of cell death can be divided into a signaling stage, followed by an effector stage, which is determined based on cellular conditions at the time and would ultimately decide the morphological type of cell death [70] – necrotic, with cell swelling and nuclear disruption; or apoptosis with chromatin condensation. This system would likely provide flexibility to modulate specific pathways depending on the signaling environment within the cell to ensure that the decision of cell death initiated by the stressor is carried out independent of modality. Thus, initial signaling could activate apoptotic death pathways, but the signaling cascade could be blocked due to parallel changes occurring in the cellular milieu, for example, inability to activate essential caspase enzymes. In such a scenario, provided the initiator signal is still ongoing, alternate pathways are activated which shift cell death to necrosis. One such established molecular switch which has been investigated recently is the receptor interacting kinase (RIP) 3, which switches cell death from apoptosis to necrosis [71] and is a critical feature of programmed necrosis. We initially demonstrated the involvement of RIP3 activation and its essential role in APAP-induced necrosis [72]. Unlike RIP1 inhibition, which exacerbated Concanavalin A induced liver injury, RIP3 inhibition consistently protected against both Con A and APAP hepatotoxicity [73] and a RIP3 specific inhibitor was shown to protect against APAP-induced hepatic necrosis in both human hepatocytes and in the *in vivo* mouse model [74]. Thus, there is clear evidence at the molecular level for a preference for the necrotic death pathway after APAP, though early features of apoptosis, such as mitochondrial Bax translocation and cytochrome c release, are also evident. However, the question could arise why cells do not undergo apoptosis once cytochrome c release occurs from mitochondria after APAP overdose. The typical mitochondrial pathway of apoptosis requires formation of the apoptosome complex by cytochrome c, APAF 1, and procaspase 9, which then activates caspase 3 to carry out apoptosis [2]. However, activity of caspase-3 was not increased at any time after APAP [14]. It should also be noted that APAP inhibited Fas-induced apoptosis only when mitochondrial damage prevented the amplification pathway and not merely when NAPQI is formed; indicating that NAPQI formation per se does not prevent pro-caspase processing and activation [14]. The lack of apoptosome formation after cytochrome c release could occur due to significant changes in the cellular milieu induced by APAP overdose, which include changes in heat shock proteins as well as ATP content as described earlier. APAP overdose has been shown to induce the upregulation of heat shock proteins such as Hsp70 in the liver [75,76] and Hsp70 interacts with the CARD domain of APAF-1 directly to inhibit procaspase-9 recruitment and apoptosome formation [77]. Probably of more relevance, induction of apoptosome formation after cytochrome c release requires dATP [78], and substantial drops in hepatocyte ATP levels have been demonstrated after APAP [39,79]. The relevance of cellular ATP in shifting APAP induced cell death to necrosis is also implicated in the aforementioned example where blocking the APAP-induced mitochondrial oxidant stress by Mito-TEMPO, which presumably preserves mitochondrial function and cellular ATP stores resulted in a limited number of cells dying by apoptosis [69]. This also illustrates the concept of redundant cell death pathways to ensure that block of one pathway would leave other options for cell death open. Thus, the mode of cell death in APAP overdose illustrates the redundant pathways which could be activated in hepatocytes. In addition, the identification of the critical role of RIP3 in influencing necrosis after APAP establishes the molecular antecedent for necrosis being the predominant form of cell death after APAP overdose.

## 7. APAP-Induced Cell Death in Hepatoma Cell Lines

In contrast to primary hepatocytes, hepatoma cell lines such as HepG2, Hep3B, SK-Hep1, HuH7 cells, and others can undergo apoptotic cell death when exposed to APAP [80-83]. However, this generally requires prolonged exposure to higher levels of APAP. The process depends on caspase activation, mitochondrial Bax translocation, mitochondrial cytochrome c release, and internucleosomal DNA fragmentation and can be inhibited by Bcl-XL overexpression [80-82]. However, the fundamental problem is that these hepatoma cells do not express any relevant levels of cytochrome P450 enzymes [84], which means that the key initiating events in the toxicity seen *in vivo*, i.e., the formation of reactive metabolites, GSH depletion, protein adduct formation, and mitochondrial dysfunction, are absent or very limited [85]. Furthermore, NAC, the only clinical approved antidote, does not protect against APAP-induced apoptosis in HepG2 or Hep3B cells [83,86]. This means that hepatoma cell lines are not clinically relevant models to study mechanisms of APAP-induced cell death *in vitro*. However, there are exceptions. HepG2 cells transfected with an adenovirus containing human CYP2E1 cDNA have been shown to have APAP-induced protein adduct formation [87]. Although these transfected cells are more susceptible to APAP toxicity and some cells die by necrosis, caspase-dependent apoptosis still appears to be the main mode of cell death [87]. More relevant to the study of drug-induced hepatotoxicity are HepaRG cells, which express a wide variety of drug-metabolizing enzymes and transporters [88] and have been shown to develop necrotic but not apoptotic cell death [85]. Importantly, most mechanisms of cell death *in vivo*, including GSH depletion, protein adduct formation, oxidant stress, and mitochondrial dysfunction contribute to APAP-induced cell death in HepaRG cells [85] similar to primary human hepatocytes [89]. Thus, the hepatoma cell line HepaRG is a relevant model for human APAP toxicity or drug-induced cell death in general [85,90,91].

More recently, the L-02 cell line has been increasingly used, especially in studies assessing the efficacy of natural products in the APAP toxicity model *in vitro* [92-95]. L-02 cells are immortalized normal human hepatocytes, which show a higher expression of Cyp2E1 than Hep3B cells [92]. Exposure of L-02 cells to various concentrations of APAP caused dose-dependent cell death involving Cyp2E1 expression, GSH depletion, JNK activation and mitochondrial translocation, mitochondrial Bax translocation, and AIF release ultimately resulting in cell necrosis indicated by ALT release and propidium iodide staining [93]. However, there are an increasing number of reports where it is claimed that APAP causes apoptosis in these cells [92,96]. Some of the conclusions are based on Bax, Bcl-2, and caspase-3 expression and other apoptosis markers with questionable specificity [94,96]. However, others show cleaved caspase-3 and DNA laddering [92]. None of the studies conclusively demonstrated the role of apoptosis in this cell line; however, if there is significant apoptotic cell death, this again would contradict studies in primary human hepatocytes [89] and in patients [97], decreasing its relevance in the clinical scenario.

## 8. Apoptosis or Necrosis – Does it Matter?

As discussed, APAP-induced cell death is clearly not an apoptotic process but has to be defined as necrosis (Table 1). This applies to APAP toxicity in isolated mouse hepatocytes [98,99], the metabolically competent human hepatoma cell line HepaRG [85], primary human hepatocytes [89], mice *in vivo* [15], and patients [97]. Defining a cell death as apoptosis versus necrosis is not just a label but the mode of cell death like apoptosis assigns a certain signaling mechanism. If apoptosis would be the dominant cell death mode in APAP hepatotoxicity, we would have effective drugs. Pan-caspase inhibitors are suicide substrates and as such are highly effective in irreversibly blocking any activated caspase in the cell within minutes after injection [60]. However, potent pan-caspase inhibitors are not protective in animal models relevant for the human pathophysiology of APAP-induced liver injury [14,15,17,66] and similar to the animal models, there is no evidence of relevant caspase activation during APAP toxicity in human cells or patients [89,97]. Thus, measuring parameters such Bax and Bcl-2 mRNA or protein expression or using the TUNEL assay and concluding there is apoptosis is scientifically unsubstantiated and incorrect. Importantly, it also bears the risk that others use these non specific parameters and questionable reasoning and thus perpetuate these wrong mechanistic conclusions. Overall, this is not only a diversion that wastes valuable resources but it also inhibits scientific progress in areas which may identify new therapeutic targets that have a chance to impact the disease. Inhibition of apoptosis will never be a realistic therapeutic intervention strategy in APAP-induced liver injury and acute liver failure.

**Table 1:** Comparison of signaling mechanisms of cell death in apoptosis and APAP-induced necrosis

Parameter	Apoptosis	APAP-induced necrosis
Bid Cleavage	Yes, Caspase mediated	Yes, Calpain mediated?
Bax translocation to mitochondria	Yes	Yes
Mitochondrial permeability transition	Yes	Yes
Release of mitochondrial cytochrome c	Yes	Yes
Assembly of Apoptosome	Yes	No
Caspase activation	Yes	No
DNA fragmentation	Yes, mediated by CAD	Yes, mediated by EndoG/AIF

## 9. Apoptosis and APAP Hepatotoxicity – Why is it a Problem?

Why do an increasing number of authors feel the need to add questionable apoptosis parameters to their papers and draw the obviously wrong conclusions that there is significant apoptotic cell death in APAP-induced liver injury? This may be part of a broader problem in science today where technology allows the measurement of more parameters in a given sample than ever before. This has led to an inflation of the amount of data presented in manuscripts with many parameters being just fillers without being really needed for the investigation. However, this resulted in the impression for many authors that more data makes a better paper and a lot of data, even though some of it may be irrelevant, is necessary to get a paper published in higher impact factor journals. The inclusion of questionable apoptosis parameters in many studies of APAP toxicity needs to be seen in this context, i.e., most authors seem to include these parameters and others just to add more data without a clear and justifiable rationale. Most importantly, there is rarely if ever a discussion regarding the relative importance of apoptotic versus necrotic cell death despite the fact that contradictory evidence for both forms of cell death are presented. This brings up the issue of peer-review, although a detailed discussion is outside the scope of this paper. Based on our own experience with reviewing a combined number of more than 200 of these types of manuscripts per year, some of them multiple times from different journals, we can conclude that many papers that claim apoptotic cell death in APAP toxicity have been rejected at least once or even multiple times before they found a home. This means that the authors received comments and suggestions for improvement from multiple reviewers. Again, based on our own observations comparing the ultimately published manuscript with earlier submitted versions, which are rarely substantially different, many authors seem to ignore the comments, no matter how valid, and shop around until they find reviewers that accept their paper. What is the harm in having flawed papers published? Science should self-correct. The experience with apoptosis and APAP clearly shows that this may not be the case anymore. If the reference lists from these types of papers are compared, it is quite obvious that these poorly designed papers are mostly copying other flawed studies. Thus, the problem is continuously perpetuated with the result that if this trend is not stopped, in few years the majority of papers in this field will actually claim, without sound scientific basis, that apoptosis is a key mode of cell death in APAP hepatotoxicity. In other words, we are in the process of creating an alternate universe where sound scientific reasoning is drowned out by massive numbers of correlative studies that conclude based on Bax and Bcl-2 protein expressions that APAP-induced liver injury is caused by apoptosis.

## 10. Is There a Solution to the Apoptosis Dilemma?

Solutions to this problem need to include a two-pronged approach. First, more authors need to be educated that peer-review is a system to improve manuscripts, which only works if the authors take the review comments seriously and modify the manuscript. This not only improves the chances to get the paper accepted by the next journal but also helps to make a relevant contribution to science and builds a positive scientific reputation. At present, the refusal to consider reviewers’ valid comments and just shop around manuscripts until less knowledgeable reviewers are encountered contributes to a flood of flawed papers with no scientific or clinical value and hurts the reputation of the authors.

The second approach is a change in the experimental design of the APAP hepatotoxicity studies. At the present time, most studies involve a pretreatment with a compound for 1-2 weeks, administration of APAP, sacrifice of the animals at a single time point and measurement of as many parameters as possible whether relevant or not. Depending on the parameters measured, it may be concluded that the compound is anti-apoptotic, anti-inflammatory, and an antioxidant. However, if the compound actually inhibits cytochrome P450, it will effectively protect by inhibiting reactive metabolite formation and as a secondary effect, prevent all negative downstream signaling events including oxidant stress, mitochondrial dysfunction, DNA fragmentation and cell death. In other words, every parameter downstream of drug metabolism will be normalized as a consequence of blocking the upstream events rather than the compound acting as an antioxidant or directly affecting other downstream events. Naturally, all mechanistic conclusions based on these types of correlations of various parameters with cell death will result in unsubstantiated and almost always wrong conclusions. It needs to be recognized that the overall pathophysiology of APAP-induced liver injury is a time-dependent process involving drug metabolism, early and late injury phases, inflammation, and regeneration [100,101]. A realistic assessment of the protective mechanism of any compound requires the investigation of effects of the compound on each of these different phases with their respective parameters. Only then can we obtain reliable mechanistic information and identify valid therapeutic targets that could impact human health.

### Conflicts of Interest

The authors declare that they have no conflicts of interest.
